# A Content Analysis of Health and Safety Communications Among Internet-Based Sex Work Advertisements: Important Information for Public Health

**DOI:** 10.2196/jmir.6746

**Published:** 2017-04-13

**Authors:** Julie Kille, Vicky Bungay, John Oliffe, Chris Atchison

**Affiliations:** ^1^ Providence Health Care Vancouver, BC Canada; ^2^ School of Nursing University of British Columbia Vancouver, BC Canada; ^3^ University of British Columbia Vancouver, BC Canada; ^4^ University of Victoria Victoria, BC Canada

**Keywords:** eHealth, communication, confidentiality, cross sectional studies, gender, health behavior, Internet, sex industry, sexual health

## Abstract

**Background:**

The capacity to advertise via the Internet continues to contribute to the shifting dynamics in adult commercial sex work. eHealth interventions have shown promise to promote Internet-based sex workers’ health and safety internationally, yet minimal attention has been paid in Canada to developing such interventions. Understanding the information communicated in Internet-based sex work advertisements is a critical step in knowledge development to inform such interventions.

**Objective:**

The purpose of this content analysis was to increase our understanding of the health and safety information within the Internet advertisements among women, men, and transgender sex workers and to describe how this information may be utilized to inform eHealth service development for this population.

**Methods:**

A total of 75 Internet-based sex worker advertisements (45 women, 24 men, and 6 transgender persons) were purposefully selected from 226 advertisements collected as part of a larger study in Western Canada. Content analysis was employed to guide data extraction about demographic characteristics, sexual services provided, service restrictions, health practices and concerns, safety and security, and business practices. Frequencies for each variable were calculated and further classified by gender. Thematic analysis was then undertaken to situate the communications within the social and commercialized contexts of the sex industry.

**Results:**

Four communications themes were identified: (1) demographic characteristics; (2) sexual services; (3) health; and (4) safety and security. White was the most common ethnicity (46/75, 61%) of advertisements. It was found that 20-29 years of age accounted for 32 of the 51 advertisements that provided age. Escort, the only legal business title, was the most common role title used (48/75, 64%). In total, 85% (64/75) of advertisements detailed lists of sexual services provided and 41% (31/75) of advertisements noted never offering uncovered services (ie, no condom). Gender and the type of Web-based platform mattered for information communicated. It was found that 35 of the 45 women’s advertisements were situated in personal websites and hosted details about nonsexual aspects of an appointment. Men and transworkers used Internet classified advertisement platforms with predetermined categories. Communications about sexually transmitted infections (STIs) occurred in only 16% (12/75) of advertisements with men accounting for 7. Women’s advertisements accounted for 26 of the 37 advertisements noting safety restrictions. Zero men or transpersons restricted alcohol or drug use. In total, 75% (56/75) of advertisements offered out-call services and the average minimal hourly rate ranged from Can $140/h to Can $200/h.

**Conclusions:**

The study findings contribute to understandings about the diverse platforms used in commercial sex advertisements, and how sex workers frame information for potential clients. This information affords health care providers and policy makers insights to how they might assist with promoting the health of Internet-based sex workers and their clients.

## Introduction

### Background

The capacity to advertise via the Internet continues to contribute to the shifting dynamics in adult commercial sex work [1,2] creating unprecedented opportunities for sex workers to determine how they will conduct business and allowing for greater control over their work [3]. Health-related benefits associated with Internet-based sex work, defined as the use of Internet by adults to facilitate consensual exchange of sexual services for money, include increased income, autonomy, and safety when compared with street-level sex work or working for a third party [1,4-6]. Internet-based sex workers can also, however, experience depression, isolation, violence, sexually transmitted infections (STIs), and substantial barriers in accessing and receiving health services [2,7-13].

Internationally, eHealth interventions have shown promise to promote health and safety among Internet-based sex workers [14,15]. Minimal attention has been paid in Canada, however, to developing interventions tailored to this population. The dearth of information about Internet-based sex work and the health practices and experiences of those involved may be contributing to oversights in eHealth intervention development [8,9,16].

Currently, the Internet is the primary advertisement medium used within the commercial sex industry. Internet advertisements for independent (ie, self-employed) Internet-based sex workers are in the hundreds of thousands each day on an international scale [1,17-23]. The analysis of information communicated within Internet-based sex work advertisements offers great promise to help improve our understanding of this population and the health and safety norms and expectations within the sex industry [8,10,21]. Indeed, an analysis of Internet advertisement content is a critical step in contributing to the knowledge needed to develop targeted and effective eHealth interventions.

To date, a small number of international studies have contributed to an evolving knowledge base about the content of Internet sex work advertisements. Although variation in the level of detail in advertisements occurs, preliminary evidence indicates that information about sexual services, pricing, locations, physical and interpersonal characteristics, and contact instructions are commonly included [8,13,19,21-28]. Gender variations in content have been reported suggesting that descriptions of personal spaces, personalities, and noncommercial services such as dinner and companionship are more common to women’s advertisements [19]. By contrast, personal biographical statements and information about who they provide services to (eg, men, women, and couples) appear more consistently in men’s advertisements [22]. Photographs are frequently included although many are partial images where the person’s face is not fully recognizable; a situation that may reflect concerns about confidentiality and privacy [19,27].

Much of the extant research on the content of Internet advertisements emphasizes men selling sexual services to other men with some conflicting results about their health practices [12,25,26]. In one US study, for instance, it was reported that 19% of Internet advertisements by men cited exclusive safer sex practices [24]. Conversely 90% of advertisements in a separate US project cited always engaging in safe sex and 16% included a statement of a preference for drug use during sexual activities [8]. Research concerning women Internet-based sex work advertisements and health and safety is minimal. Women’s advertisements are frequently investigated from male clients’ perspectives emphasizing clients’ discussions and reviews of sex workers embedded in Internet social networking forums [28] or analysis framed by queries about what male clients find attractive [27]. Moreover, research concerned with the content of Internet advertisements has focused on marketing strategies, sexual service information, and privacy concerns about sex workers’ identities. Less attention has been paid to the types of health, safety, and transactional information that are communicated within these advertisements.

### Purpose of the Study

In this paper, we contribute to the growing knowledge about Internet-based sex work by detailing the results of a content analysis of Web-based advertisements within a Canadian context. The study purpose was to increase understandings of the health and safety information within Internet-based sex work advertisements and to offer suggestions for how this information may be utilized to inform eHealth program development. Because people of all genders engage in sex work [29] and gender matters for health [30], the content of Internet advertisements were examined within and between subgroups of women, men, and transgender or transpersons (defined as individuals who were assigned a sex at birth but express and experience their gender differently; however, they do not necessarily undergo sex reassignment or corrective surgery) [16] engaged in sex work.

## Methods

### Overview

This study was conducted using a content analysis approach [31,32] following university ethics approval. Content analysis has been used extensively by gender scholars to compare the information noted in Internet- and media-based communications of men and women [33]. Included were a series of iterative and systematic processes that involved creating an empirically derived sample of Internet advertisements, determining the unit of analysis (eg, words, phrases, photos), developing a categorical scheme to code the data, and conducting data analysis [32].

### Sample

The sample consisted of 75 verified Internet advertisements for sexual services provided by women (n=45), men (n=24), and transgender people (n=6) advertising specifically in Vancouver, British Columbia, Canada. The empirically driven sample [32] was selected from a database of 226 Web-based Canadian advertisements of women (n=173), men (35), and transgender (n=20) workers collected during ethnographic mapping activities of a larger study examining the relationships between health, safety, and the organizational features of an indoor sex industry. Ethnographic mapping involved a series of key informant interviews to detail prominent classified websites and search strategies for personal business websites situated within the local context, visiting the websites and classifieds 2 times per week over a 4-week period to collate number of posts and strategize for duplication removal, and narrowing to 3 Web-based classified platforms that were used most consistently; the details of which are described elsewhere [20]. Purposeful sampling was used to capture the diversity in Web-based sex advertisement formats including advertisements from 35 personal websites and 40 from 3 Web-based classified advertising platforms. All advertisements were publicly accessible and membership or sign-in was not a requirement.

### Data Collection

Due to the potential temporary nature of Internet advertisements, screenshots were taken and downloaded to a password-protected folder (see Multimedia Appendix 1). Drawing from previous research on the content of information communicated within Internet-based sex work advertisements [8,12,19,21,22,25-27,34] and the study objectives, a coding scheme that included relevant, advertisement-specific variables and related operational definitions (Table 1) was used [21,33]. Each variable was also coded as a categorical “yes” or “no” to document variable presence or absence within the advertisement. Data extraction and coding were undertaken by the first two authors collaboratively to aid reliability of the coding process. Data extraction of the variables and associated measures was completed by using a Microsoft Excel file template.

**Table 1:** Variables and operational definitions.

Variable	Operational definition
Sexual services provided	Sexual service provision communications^a^ refer to the types of sexual acts that the person offers within their advertisement.
Sexual service restrictions	Restriction communications refer to the sexual services that the person advertising reports being unwilling to engage in during the provision of sexual services.
Health	Health communications were operationalized into two categories: (1) communications that specifically speak to health-related practices that could be considered protective or nonprotective for health (eg, nonsmoker, drug use, condom use) and (2) presence or absence of specific health issues (eg, HIV^b^, sexually transmitted infections).
Safety and security	Safety and security communications refer to factors associated with positively or negatively affecting people’s physical, emotional, and financial well-being including their privacy. Specific categories for extraction included (1) location where sexual services provided, (2) instructions for clients on how to prepare for the encounter, (3) security measures taken before encounter including screening processes, (4) security measures during the encounter, and (5) facial photo details.
Demographic characteristics	Demographic communications refer to salient information about the “structure” of the population and included age, ethnicity, gender, and business title (eg, escort, companion).
Business practices	Business practices refer to communications that detail the dollar amounts associated with the cost of sexual services, contact and booking processes, and related restrictions.

^a^Communication was defined as the process of sharing information and conveying meaning through a shared system of semiotic rules and symbols [35]. Hypertexts or the array of words, phrases, and images found within advertisements were the focus of data collection [32].

^b^HIV: human immunodeficiency virus.

### Data Analysis

Variable frequencies were calculated using descriptive statistics and further classified according to gender. The social and commercialized contexts in which the information was embedded were also considered [32], including norms of language, the legal status of sex work in Canada, and the diversity of sexual services and Web-based advertising formats within the marketplace [9]. Thematic analysis of the hypertexts and related frequencies were undertaken to synthesize and categorize types of information communicated and the gender similarities and differences within the social and commercial contexts of the Web-based advertisements. Four communication themes were identified: demographics, sexual services, health, and safety and security.

## Results

### Overview

The 75 advertisements varied in structure and format in how the information was communicated and organized. All the 75 advertisements marketed sex services to men and 2 men’s advertisements additionally advertised services for women clients. In total, 78% (35/45) of women’s advertisements were within their personal business websites, whereas men’s (22/24; 83%) and transpersons’ advertisements (5/6; 83%) appeared within Internet classified advertising platforms. Unlike tailored personal websites, the advertisements within the classified platforms adhered to an advertising template with information organized into predetermined categories (eg, age, weight, services offered, classification). Women’s advertisements within their personal websites frequently described their workspaces in detail with emphasis on the provision of a relaxing atmosphere. Classified platforms did not afford the opportunity to detail a space, only whether or not one was provided. Health, safety, demographic characteristics, and sexual services were regularly communicated in the 75 advertisements regardless of platform used.

### Demographic Communications

Sex industry language norms were evident in the work titles and physical characteristics communicated throughout the advertisements with variations that mapped onto gender. Although "escort" was the most common work title (Table 2) across groups, it was the only term used in the men's and transpersons' advertisements. By contrast, almost half of the women’s advertisements included other titles that evoked specific heterosexual erotic identities, such as “courtesan,” “playmate,” or “dominatrix.” The use of escort for a work classification may also be understood within the constraints of the Canadian laws that criminalize communications associated with selling and purchasing sexual services. The term “escort,” for example, is a legally recognized business title within the City of Vancouver municipal bylaws [36], whereas other titles are not. Additionally, the laws may contribute to restrictions on some of the Web-based classified platforms regarding language permitted within their site.

**Table 2:** Demographic characteristic communications.

Characteristics		Women (n=45)	Men (n=24)	Transgender persons (n=6)	Total n=75 n (%)
**Work title**					
	Escort	18	24	6	48 (64)
	Companion	5			5 (7)
	Multiple roles	4			4 (5)
	Other	10			10 (13)
	Not specified	8			8 (10)
**Age (years)**						
	20-29	14	16	2	32 (43)
	30-39	9	4	1	14 (18)
	40-49	1	3	1	5 (7)
	Not specified	21	1	2	24 (32)
**Ethnicity**					
	White	29	13	4	46 (61)
	Black	2	1		3 (4)
	Latino or Hispanic	1	3		4 (5)
	Asian	9	4	1	14 (19)
	Multiethnic	4	3	1	8 (10)
**Weight**					
	Advertisements specifying weight	25	24	4	53 (71)
	Average of specified weights (lbs)	118	167	135	

In total, 25 of the 75 advertisements did not specify the sex workers' ethnicity. In these cases, photos accompanying advertisements provided an indication from which ethnicity was estimated. White was the most common ethnicity stated or observed (Table 2). Men’s advertisements were the only ones to consistently provide age, weight, and ethnicity (Table 2), which may reflect the template of the classifieds’ advertising platforms. Two transpersons’ advertisements communicated surgical or hormone replacement therapy details such as “undergone hormone replacement therapy” or “nonoperative transsexual” highlighting the significance of language to communicate about their embodied specificities. Men’s communications detailed their muscular build or fitness, for instance, “physically fit, muscular” or “lean or swimmer build” and included physical characteristics, the most common being weight, body hair, body type, and penis size. This differed from the women and transpersons’ advertisements wherein physical descriptions such as bust size were present in some cases but did not appear uniformly.

### Sexual Service Communications

The language used to communicate about sexual services among the 64 advertisements listing sexual services reflected sex industry norms. Variation between genders and homogeneity within was observed (Table 3). The term “full service,” communicating to clients a willingness to engage in sexual activities that included vaginal intercourse was found within women’s advertisements. Women also listed the “girlfriend experience (GFE) or boyfriend experience (BFE),” which is defined as the provision of dating activities and sex that would be expected in a noncommercial adult relationship [37], more often than men. Men’s and transgender peoples’ advertisements used the terms “top,” “bottom,” or “versatile,” indicating their sexual positions for anal intercourse. Men also used the term “anal” explicitly, whereas women used the term “greek” to indicate anal services.

**Table 3:** Sexual services communications.

Characteristics		Women (n=45)		Men (n=24)		Transgender persons (n=6)		Total n=75 n (%)	
Advertisements with services listed		35		23		6		64 (85)	
**Type of services**									
	Full service		20						20 (27)
	Massage		18		18				36 (48)
	GFE^a^ or BFE^b^		17		2		1		20 (27)
	Companionship		12		12				24 (32)
	Duos		12		1				13 (17)
	BDSM		12		12		1		25 (33)
	Greek or anal		6		16		3		25 (33)
	Uncovered services		11		6		1		18 (24)
	Top (position)				6				
	Bottom (position)				6				
	Versatile (top or bottom)				11		2		
Advertisements with service restrictions		26		9		2		37 (49)	

^a^GFE: girlfriend experience.

^b^BFE: boyfriend experience.

The language of men’s advertisements was further associated with terms that are used to describe specific sexual practices between men, for instance, “rimming, fisting, and watersports,” and included detailed lists of sexual acts that they would engage in. Diverse BDSM services were listed across genders. BDSM is a term used to denote varied consensual sexual practices that include bondage, domination or submission, pain or sensation play, power exchange, leathersex, role playing, and fetishes [38]. The women’s advertisements offering BDSM services additionally communicated information about health and safety. Clients were asked to provide information about any medical issues and a safe word to signal when they wanted to stop the activity before services. Women’s advertisements more often identified and provided hyperlinks to the websites of other men and women sex workers with whom they worked as part of their “duo” services (Table 3).

Communications about condom use included the terms “covered” (ie, condom) and “uncovered” (ie, no condom required) with service variations noted among the 64 advertisements listing services. Uncovered services were usually specific to oral-genital sex (Table 3). Women used industry acronyms such as BBBJ (bareback blowjob), CIM (cum in mouth), DATY (dining at the Y/cunnilingus), and DATO (dining at the O/analingus) to detail the service. Uncovered analingus or “rimming,” was offered in 6 of the 23 men’s advertisements listing services. In contrast, 41% (31/75) of advertisements stated that they *never* provided uncovered services, often describing their services as “always safe” (see Multimedia Appendix 2). Women communicated more restrictions overall, sometimes posting an “etiquette” or “frequently asked questions” section that outlined restrictions.

### Health Communications

There were noteworthy gender-based variations in the Web-based information about personal health practices. Transworkers’ advertisements rarely communicated any personal health information, whereas men and women consistently provided health-related details. Communications about cigarette smoking and substance use were the most common types of personal health practice communications (Table 4). It was found that 7% (3/45) of women’s advertisements also contained communications requesting clients refrain from smoking before or during appointments, and one of the women’s advertisements indicated that she would not visit smoking rooms in hotels.

Although none of the men or transgender persons explicitly restricted clients’ drug or alcohol use, 22% (10/45) of women’s advertisements stated a refusal to see a client who was, or appeared to be, under the influence of drugs or alcohol. It was found that 25% (6/24) of men’s advertisements stating that they *did* use drugs noted being okay with “party and play” (PNP), a euphemism for acknowledgment of the availability for drugs, frequently crystal methamphetamine, to be taken in conjunction with having sex [39]. None of the advertisements stated specific illicit drugs with the exception of a 2 women’s advertisements that specified being “420 friendly” indicating marijuana was acceptable.

Communications about STIs were infrequent (12/75, 16%) and higher among men’s advertisements. “Clean” and “infection-free” were the terms used. One of the men’s advertisements communicated that he was “HIV-positive” but “undetectable,” a status referring to HIV viral load as measured through serum testing [40]. His advertisement further noted that although bareback was preferred, he would provide covered services. Client hygiene was commented upon in women’s advertisements only. Expectations of “good hygiene” were noted and they also included information about the facility to shower at their in-call before services (see Multimedia Appendix 2).

**Table 4:** Health communications.

Characteristics		Women (n=45)	Men (n=24)	Transgender persons (n=6)	Total n=75 n (%)
Health communications		29	24	1	64 (85)
**Sex workers’ personal health behaviors communications**					
	Nonsmoker	16	9		25 (33)
	Nondrug using	13	3		16 (21)
	“Social” alcohol use	7	4		11 (15)
	Uses drugs	2	6		8 (11)
	Other (physical activities such as yoga, gym)	3	10	1	14 (19)
Communicable infection communications		5	8		12 (16)

### Safety and Security Communications

Communications associated with personal and financial safety and security occurred in all advertisements and were regularly embedded in information for potential clients to learn about the operational features of connecting with and making an appointment with a worker (Table 5). For example, in addition to detailing the location where appointments would be held, specific contact instructions were provided. Multiple options for making contact were consistently available across genders (Table 5); however, the patterns of use varied with men and transpersons’ advertisements more frequently using the messaging features of the hosting classified advertisement platform. Women utilized a live (ie, phone) booking process with potential clients. It was found that 9% (7/75) advertisements noted not accepting blocked numbers for phone calls, of which 5 were in women’s advertisements.

The advertisements also illustrated that Internet-based sex work occurs within in-call spaces such as apartments provided by the sex worker, and out-call locations where a sex worker travels to the client. Although there were limited restrictions communicated concerning out-call locations, gender variance was observed with only women communicating that they would not provide out-call services to private residences, or in hotels rated below a 4-5 star accommodation. It was found that 9% (4/45) of the women required references from another service provider and one woman required clients to check-in with hotel security before the exchange. None of the transworkers shared any communications about willingness to travel; although just under one-third and one-quarter of men and women’s advertisements, respectively, noted this as an option.

Attempts to maintain privacy and security were also reflected in the photos provided within the advertisements. One-third of advertisements contained identifiable facial photos, including all of transpersons’ advertisements, and one-half provided a viewable photo of some kind (Table 5). The partial face photos revealed features such as eyes or lips, or in some cases a side profile with shadowing that made it more difficult to identify the person in the photo. In total, 2 of the 45 women’s advertisements explicitly noted that clients were not to take photos or videos during an appointment and one woman’s advertisement stated that she would agree to photos and videos only if she was confident that they would never be posted on the Internet, but she did not state how that would be negotiated.

The communications about pricing provided concise information about fees. The price of service varied with women charging on average approximately 25% less than men per exchange (Table 5). Admittedly, the highest rate in the sample, Can $700/h, was listed on a woman’s site; however, the advertisement was an outlier in terms of fee. The men and transworkers’ sites listed consistent prices per hour; and both men and women charged significantly more for travel and did not include the price of airfare, hotels, and meals, which was specified separately.

**Table 5:** Safety and security communications.

Characteristics		Women (n=45)	Men (n=24)	Transgender persons (n=6)	Total n=75 n (%)
Safety and security restrictions for client behaviors identified		26	9	2	37 (49)
**Service locations**					
	In-call	35	12	3	50 (67)
	Out-call	34	18	4	56 (75)
	Willing to travel out of town	9	7		16 (21)
**Viewable facial photo**		22	11	5	38 (51)
	Full	13	9	5	27 (36)
	Partial	9	2		11 (15)
**Booking contact process**					
	Phone	35	15	5	55 (73)
	Website	15	19	3	37 (49)
	Text	8	1	1	10 (13)
	Email	18	5	9	32 (43)
**Rates (Canadian dollar)**					
	Minimum	140	180	200	
	Maximum	700	450	250	
	Average	313	247	233	

## Discussion

### Principal Findings

The study findings build on nascent empirical understandings of the content of Web-based advertisements [19,22,23,27,41]. This research also contributes particular topics for consideration in public health and eHealth programming among the growing population of sex workers who use the Internet as their primary means to communicate, at least initially, occupational health and safety details to potential clients. In these advertisements, sex workers communicated significant information that reflected their personal health practices both within and outside commercial sex exchanges (eg, drug and alcohol use, condom use, physical fitness). These health communications provide important insights to the nature and norms of Internet-based sex work and the practices, health behaviors, and overall wellbeing of those advertising in this context.

The findings also illustrate that gender matters when considering health and safety practices and Internet-based communications. Women list more safety communications overall and do so by communicating restrictions, particularly about place and substance use. Women also use personalized screening processes involving live phone communications to determine the suitability of the client [5]. Men and transpersons communicate minimal safety details. These finding adds nuance to our understandings about different strategies between men and women sex workers to mitigate risks of violence [13,42,43] by demonstrating the role of Internet-based communications within the realm of the commercialized exchange. Gender and technology also intersect to influence the content of communications including how information is presented, language used, and the actual types of details included. Women tend to use personal business websites to advertise providing substantial details about etiquette and atmosphere. Men and transpersons use Web-based classified platforms with predetermined categories and emphasize physical characteristics. Understanding the gender nuance of advertising platforms is essential to developing targeted eHealth messaging that takes into consideration the norms of communication within the realm of the diverse platforms used [1,18].

The findings also provide some new insights into population characteristics within Canadian advertisements situated in this locale as predominantly white, young workers who charged over Can $200/h, on average, for their services in both in-call and out-call settings. These characteristics are similar to those described in other North American Internet-based sex work research [27,44] and in direct contrast to research situated in street-based marketplaces that demonstrate higher numbers of Indigenous, Black, and Hispanic people aged over 30 years and diverse financial arrangements where fees charged are amount per service with much lower income potential [25,45]. As expected, the snapshot of the Internet-based sex industry captured in these 75 advertisements also indicates that men, women, and trans-sex workers provide services almost exclusively to male clients.

The health communications regarding condom use raised several important issues warranting attention. Condom use was regularly communicated for anal or vaginal intercourse, yet less than half of the advertisements communicated that a condom was required for oral-genital sex. Although there is growing evidence that many sex workers may experience STI infection rates similar to or less than the general population [12,46,47], the lack of condom requirement for oral-genital sex may still pose a significant health threat for them [41,48,49]. For example, STIs such as syphilis, gonorrhea, and chlamydia can be transmitted via oral-genital contact and can be asymptomatic [50]. Women aged under 30 years and men having sex with men are disproportionately affected by some STIs [51]; an important fact given the demographic characteristics of the study sample. Moreover, there is some evidence that sex workers, like many in the general population, may not fully comprehend the possibilities of transmission of STIs through oral-genital body fluid exchange [52,53].

Developing health promotion strategies for condom use, however, requires nuanced understandings of evolving commercial sex industry norms. Men and women Internet-based sex workers are engaged in offering emotionally erotic exchanges for sale and purchase that are designed to foster intimacy and replicate aspects of noncommercial relationships [54,55]. Additionally, the demand for unprotected sexual services is steadily on the rise and is directly contributing shifting norms and economic influences shaping safer sex practices [47,53]. It has been demonstrated, for instance, that women’s willingness to engage in uncovered oral sex is associated with increased earnings offered by clients for those services irrespective of their health-related knowledge [53]. How the issues of knowledge, intimacy, companionship, economy, and shifting norms influence people’s practices, their health and safety highlights the need for contextually appropriate approaches to sexual health promotion and STI prevention. Contextually appropriate approaches that take into consideration the multiple cofactors that can contribute to STI transmission and the diverse needs that people may have with regards to building prevention capacity have been shown effective in diverse populations [56]. It is, therefore, critical that public health consider how to target multiple interrelated issues such as money, intimacy, and familiarity in the campaigns aimed at promoting sexual health and people’s safer sex behaviors.

Finally, Internet-based sex workers are often considered a hard-to-reach group particularly because their businesses operate in limitless Web-based spaces afforded by Internet-based advertising services [20]. Isolation and barriers in access to health promotions services have been noted among this population [7-13]. eHealth programs delivered via the Internet are often successful because of their potential to reach people who experience isolation [57]. To be effective, however, it is essential that such health programming be developed to reach the diversity of sex workers and to do so, programming must involve the active participation of those engaged in sex work in the design and implementation [11,58,59]. As the World Health Organization (WHO) strategy for the health promotion of sex workers noted, “health services should be made available, accessible, and acceptable to sex workers” (p.xix). Moreover, health promotion programs that build upon the existing strengths and the knowledge base of sex workers have a proven track record as the most successful to promote health and safety [11]. Additionally, health promotion strategies must include and extend beyond STI prevention. The need for greater knowledge and skills in the areas of safe and effective Internet advertising strategies, attracting and screening appropriate clients, addressing legal concerns, and financial planning—an important aspect of transitioning out of the industry have been identified [44,60]. Given the evidence of increased autonomy and independence associated with Internet-based sex work [4-6], there is perhaps an opportunity for health programming to capitalize on the benefits of autonomy and control in sex workers’ choice to communicate explicit Web-based restrictions, and to promote and expand self-health in the sex industry as a highly desirable and marketable characteristic.

### Limitations

The research is limited by the small sample and the limited information gleaned from the transworkers’ advertisements. And, although content analysis provides some information to help understand health and safety practices, additional research with a larger sample that includes the perspectives and experiences of Internet-based sex workers is warranted.

### Conclusions

This descriptive study is one of the first to analyze the content of Internet-based sex work advertisements in Canada, and is one of a few to include men, women, and transgender people in a single study. Content analysis is a feasible approach to understanding Internet-based communications and must be nuanced within the social and commercialized context of the sex industry to help inform health service programming for Internet-based sex workers [44]. Strategies for eHealth promotion must be informed by more relational approaches to sex and intimacy that are inclusive of sex workers and their clients and considers gender-specific aspects of these relationships. As the Internet continues to grow, greater numbers of sex workers will likely advertise services via the Internet. Important insights can be gleaned by describing Internet advertisement content about industry practices including the emergence of self-management akin to small business ownership. With this in mind, we might thoughtfully continue to describe Internet-based sex workers’ advertisements as a means to developing more effective eHealth programming to better support the health of sex workers and their clients.

## Appendix

Multimedia Appendix 1 Sample screenshots from a personal business website and a Web-based classified advertising site.

Multimedia Appendix 2 Additional advertising details are provided in table format concerning communications about restrictions, infections, and rules for client health behaviors.

## Abbreviations

BBBJ: bareback blowjob
BFE: boyfriend experience
CIM: cum in mouth
DATO: dining at the O/analingus
DATY: dining at the Y/cunnilingus
GFE: girlfriend experience
PNP: party and play
STI: sexually transmitted infection
WHO: World Health Organization
