# Supplementary material for: A Content Analysis of Health and Safety Communications Among Internet-Based Sex Work Advertisements: Important Information for Public Health
Source: J Med Internet Res. 2017 Apr 13;19(4):e111. doi: 10.2196/jmir.6746 (PMC5408134; doi:10.2196/jmir.6746)

Multimedia Appendix 1: Sample Screenshots

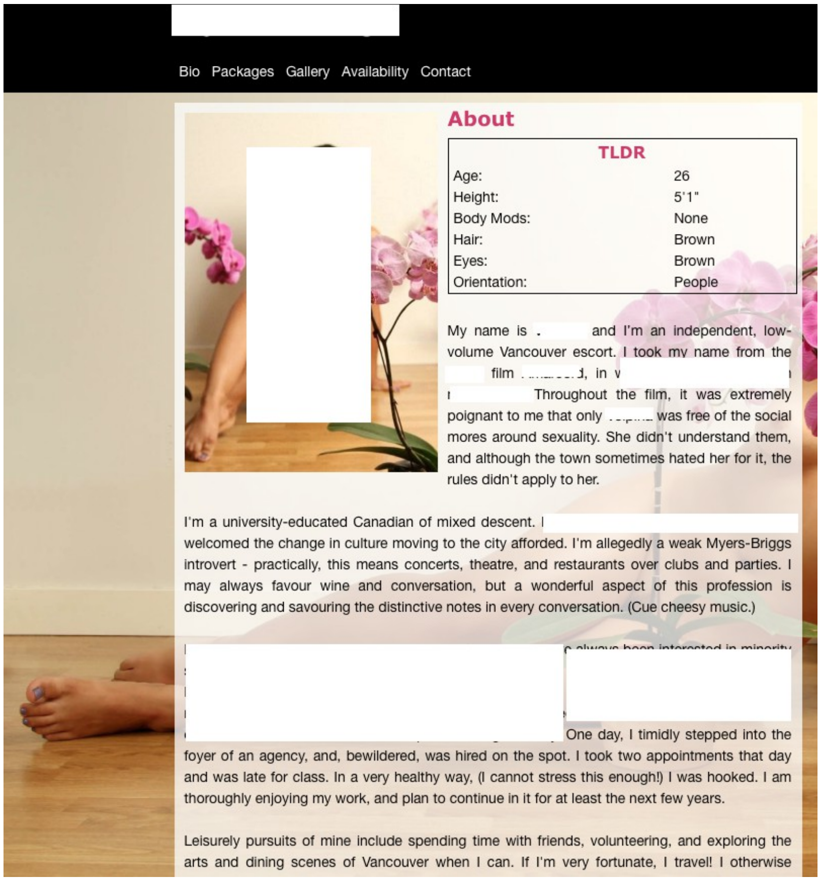

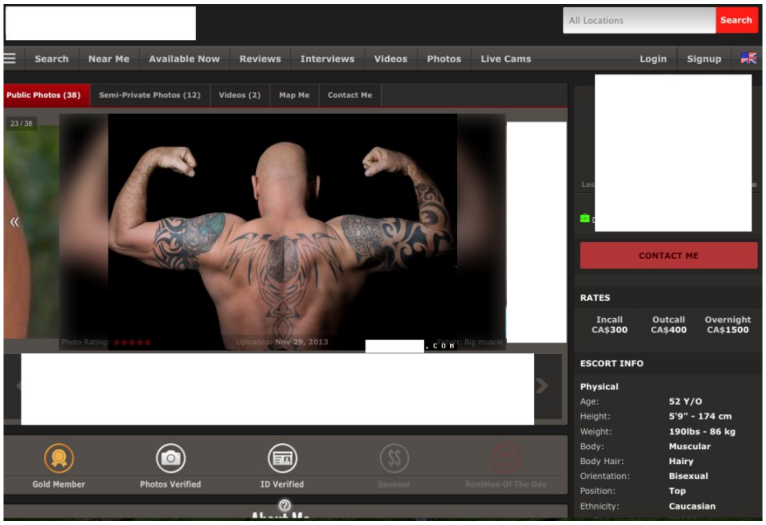

Supplement: Multimedia Appendix 1 [file jmir_v19i4e111_app1.pdf]
