# Supplementary material for: A Content Analysis of Health and Safety Communications Among Internet-Based Sex Work Advertisements: Important Information for Public Health
Source: J Med Internet Res. 2017 Apr 13;19(4):e111. doi: 10.2196/jmir.6746 (PMC5408134; doi:10.2196/jmir.6746)
Supplement: Multimedia Appendix 2 [file jmir_v19i4e111_app2.pdf]

Multi Media Appendix 2: Additional Advertising Details

Characteristic	Women (n=45)	Men (n=24)	Transgender persons (n=6)	Total n=75 n (%)
**Ads with Services Listed**	35	23	6	64 (85)
**Types of Service Restrictions**				
Uncovered services	21	9	1	31 (41)
Greek/anal	9			9 (12)
Light-deep French kissing	5			5 (7)
Other physical restrictions	7	1	1	9 (12)
**Communicable Infection Communications**	5	8		12 (16)
Do not have any STI	5	7		12 (16)
Have an infection		1		1 (1)
**"Rules" for Clients' Health Behaviors**				
OK with using drugs during encounter	2	6		8 (11)
Require client to shower at the location prior to service	5			5 (7)
**Safety and Security Restrictions for client behaviors identified**	26	9	2	37(49)
Blocked numbers	5	1	1	7 (9)
Drinking/appearing drunk	10			10 (13)
Taking pictures or videos during the encounter	2			2 (3)
Other	9	8	1	18 (24)
Unwilling to attend private residence	3	1	1	
